# Adaptive Data Boosting Technique for Robust Personalized Speech Emotion in Emotionally-Imbalanced Small-Sample Environments

**DOI:** 10.3390/s18113744

**Published:** 2018-11-02

**Authors:** Jaehun Bang, Taeho Hur, Dohyeong Kim, Thien Huynh-The, Jongwon Lee, Yongkoo Han, Oresti Banos, Jee-In Kim, Sungyoung Lee

**Affiliations:** 1 Department of Computer Science and Engineering, Kyung Hee University, (Global Campus), 1732, Deogyeong-daero, Giheung-gu, Yongin-si, Gyeonggi-do 17104, Korea; jhb@oslab.khu.ac.kr (J.B.); hth@oslab.khu.ac.kr (T.H.); dhkim@oslab.khu.ac.kr (D.K.); thienht@oslab.khu.ac.kr (T.H.-T.); jwlee2oo@hanmail.net (J.L.); ykhan@khu.ac.kr (Y.H.); 2 Department of Computer Architecture and Computer Technology, University of Granada, C/Periodista Daniel Saucedo Aranda s/n, E-18071 Granada, Spain; oresti@ugr.es; 3 Department of Smart ICT Convergence, Konkuk University, 120 Neungdong-ro, Gwangjin-gu, Seoul 05029, Korea; jnkm@konkuk.ac.kr

**Keywords:** speech emotion recognition, personalization, machine learning, data selection, data augmentation

## Abstract

Personalized emotion recognition provides an individual training model for each target user in order to mitigate the accuracy problem when using general training models collected from multiple users. Existing personalized speech emotion recognition research has a cold-start problem that requires a large amount of emotionally-balanced data samples from the target user when creating the personalized training model. Such research is difficult to apply in real environments due to the difficulty of collecting numerous target user speech data with emotionally-balanced label samples. Therefore, we propose the Robust Personalized Emotion Recognition Framework with the Adaptive Data Boosting Algorithm to solve the cold-start problem. The proposed framework incrementally provides a customized training model for the target user by reinforcing the dataset by combining the acquired target user speech with speech from other users, followed by applying SMOTE (Synthetic Minority Over-sampling Technique)-based data augmentation. The proposed method proved to be adaptive across a small number of target user datasets and emotionally-imbalanced data environments through iterative experiments using the IEMOCAP (Interactive Emotional Dyadic Motion Capture) database.

## 1. Introduction

Recently, various machine learning techniques, such as representation, translation, alignment, fusion, and co-learning, have been researched for multimodal user interfaces, analyzing various sensor information, such as text, image, video, and sound [1]. The multimodal user interface research has been progressed to recognize emotion from video information that includes audio images using the latest deep learning technology, the convolutional neural network [2]. In particular, speech information is an important information that naturally recognizes emotions, and emotion recognition techniques using various machine learning strategies and algorithms are under study.

Various technologies, such as audio preprocessing, feature extraction, model creation, feature/decision level fusion, and adaptation, have been researched in speech emotion recognition recently [3]. Traditional speech emotion recognition studies aim at improving the feature extraction and classification methodologies to improve the accuracy of various amounts of recorded emotional speech from multiple users. Such feature extraction studies consist of filter-bank algorithm improvements and statistical feature discoveries [4,5]. On the other hand, the classification studies include a hierarchical classification methodology [6], a mixture of two classifiers [7], and the creation of training models of males and females [8]. These previous studies achieved high accuracy based on speaker-dependent (SD) model experiments, where the users participated in the training process. However, the accuracy is significantly lowered when the target user’s speech does not participate in the training [9].

Therefore, the speech emotion recognition studies have been conducted to create a training model that achieves high accuracy in speaker independent (SI) experiments. SI model studies also have been researched to create a highly accurate predictive model for every user. The accuracy of an SI model was lower than that of existing SD models given the same amount of training data. Nowadays, the gap of accuracy difference has been reduced with the SD model by introducing many machine learning techniques and strategies, such as deep learning [10,11], extreme learning machines [12,13], classification fusions [14], and Adaboost MH (Multi-class Hamming trees) [15]. These methods have the advantage of providing a recognition service to users with reasonable models immediately in the initial stage. However, these methods still do not guarantee the recognition of emotions with high accuracy levels for every user. Additionally, the SI model also requires a sufficient training dataset in training phases to achieve reasonable accuracy. Furthermore, it is difficult to improve the accuracy level due to using a static training model.

Recently, speech emotion recognition research has focused on creating a personalized model that can provide a high accuracy level to every user by providing customized dynamic models to each user. The important thing of creating a personalized model is how to provide recognition services to the target user with a reasonable accuracy. In personalized emotion recognition, the target user’s real speech data is required. The personalized model is most affected by target user speech data. In the field of speech based emotion recognition, the collected data is the most influential, and the training model should be modified to the maximum extent as possible.

In the personalized emotion recognition process, the initial model should also be generated in a limited dataset environment, where there are small samples of the target user’s dataset. On the other hand, it is very important to establish an efficient machine learning strategy to create an accurate personalized model in the whole life cycle of the recognition process.

There are three typical machine learning strategies, convolutional learning, self-learning, and adaptive learning. The convolutional learning strategy is to improve the accuracy level by extracting a various and sophisticated feature set from the obtained large scale data set [16]. This strategy requires a lot of reference datasets in the feature vector extraction process to build an accurate training model in various feature schemes. Therefore, it is difficult to find the combination of feature vectors suitable in an environment where there is not much target user data [17].

The self-learning strategy is a system in which the system automatically changes the existing general model by continually adding user data [18,19]. Even in this strategy, when the target user’s speech data has a small number, the influence of the existing training model is much larger than personal data. Therefore, too much target user speech is required to change the personalized model from the existing model.

The adaptive learning strategy method involves some intervention by the user and guarantees a high degree of accuracy through direct modification of the training model. This strategy is performed with high accuracy when acquiring a large scale target user dataset as time goes on [20]. Additionally, this strategy can improve the accuracy in the initial stage by changing the model directly.

The convolutional and self-learning strategies are optimized in a large dataset environment to achieve high accuracy. However, the personalized system cannot acquire the large scale of target user’s data samples in the initial stage. Therefore, these two strategies face underfitting problems exploiting the small target user’s data set. In other words, these strategies cannot modify the training model rapidly due to the method of adding new data to the existing training model.

However, the adaptive learning strategy can avoid the underfitting problem through training data augmentation or combining the existing dataset with the target user’s dataset from feedback. In other words, this strategy can modify the training model directly to solve the bias problem in small datasets, such as modifying the boundary of the model. Therefore, it is an effective method in the field of personalized emotion recognition, if the user’s intervention is minimized and the personalization factor can be accurately considered [21]. Thus, most of the personalized emotion recognition has researched the speaker adaptation (SA) model using adaptive learning strategies when considering the amount of limited data and the duration of the training process.

SA models are dynamic training models for target users created by combining the target user speech with user speech from multiple users. SA model research consists of feature normalization, supervised adaptation, and unsupervised adaptation. Feature normalization studies [22,23] have created personalized models through iterative feature value normalization processes. In particular, these models can create individual models for target users by controlling the overall ranges of the feature values of the training dataset. However, in small-sample environments, these studies have not achieved high accuracy, as it is difficult to estimate target user speech characteristics. Supervised adaptation studies [24,25] consist of individual model creation utilizing only the target user speech and incremental learning [26,27], which adds target user speech to existing multiple-user training models. However, these methods require large amounts of data to create personalized models that are dependent on the target user speech. Unsupervised adaptation [28] has an advantage in easily constructing SA models via cluster models of the target user speech without any emotional speech annotation processes. However, this leads to lower accuracy when using small amounts of samples, making it difficult to predict the probability distribution of clustering.

In other words, the experimental results of existing SA studies have considered numerous target user samples and balanced data for each emotion. In real environments, the acquired target user speech in the initial stage cannot guarantee a large number of samples with balanced emotion due to imbalanced emotion expression as seen in daily life. Regarding the small amount of imbalanced data at the initial stage, the experimental results indicate that no reinforcement methods have been conducted due to the lack of emotional speech cases. This is known as a cold-start problem, which can be overcome by constructing personalized training datasets using real data selection and virtual data augmentation.

Therefore, we propose the adaptive data boosting (ADB) method to deal with the cold-start problem in small and imbalanced datasets during the initial stage and implement the robust personalized speech emotion recognition framework. The proposed ADB reinforces the training dataset with a similar real training data when there is an insufficient amount or absence of emotion data. This process is conducted by constructing a similarity of speech feature vector by comparing the acquired target user speech with the initial multiple-user database. Further, we also augment virtual data using the SMOTE (synthetic minority over-sampling technique) algorithm to create a robust model considering the new data. The proposed personalized speech emotion recognition framework incrementally provides personalized models for target users through a retraining process via a machine learning algorithm based on the boosted personalized data from ADB.

## 2. Robust Personalized Emotion Recognition Framework

The framework introduced in this section incrementally creates an acceptable training model using a minimal number of target user samples via the proposed adaptive data boosting methods. This framework is an innovative system that can resolve the cold-start problem present in small and emotionally-imbalanced data environments. The proposed ADB, which is the core methodology of this framework, consists of data reinforcement and data augmentation. The data reinforcement method selects real data by determining the similarity of speech datasets between the acquired target speech and the initial multiple-user training model. The data augmentation method generates virtual data to create more scenarios by utilizing SMOTE. The boosted data extracted via the ADB process constructs the personalized training model using a machine learning algorithm.

**Figure 1:**
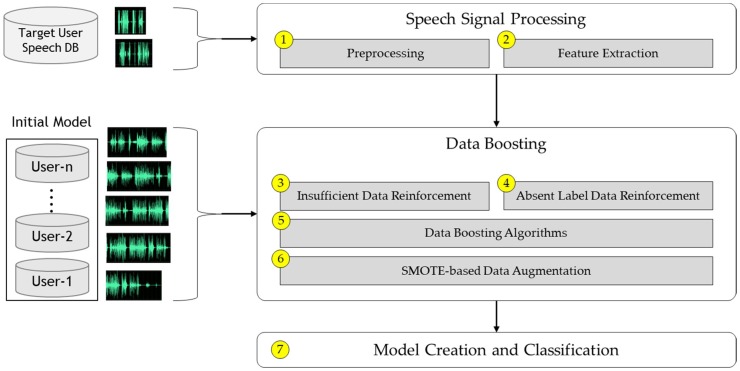
Proposed robust personalized emotion recognition framework.

This framework can create and update a personalized model incrementally for a target user by implementing a re-training process with only a single target user input. Figure 1 shows the system architecture of the proposed method.

**① Preprocessing**

**Figure 2:**
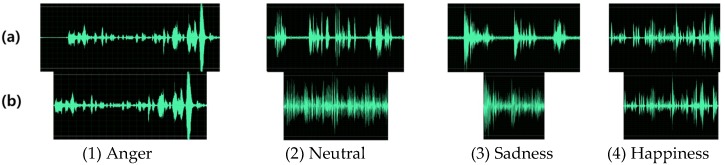
Waves of (**a**) before and (**b**) after preprocessing module in a sentence.

This module performs normalization and the silence removal process. We employed the peak normalization implemented by jAudio [29], which is the default approach to adjusting the data value based on the highest signal level present in the audio. Additionally, we also employed the existing silent removal approach based on the zero crossing rate (ZCR) for speaker identification [30] to discard the blank area in the speech. This approach divides audio into frames, where each duration is segmented in 15 ms by a hamming window. Then, speech boundaries are estimated based on the short time energy (STE) algorithm. After that, silence areas are removed by the zero crossing rate value. This method can extract user’s speech in consideration of the noise level. Figure 2 presents the examples of waves of before and after applying the silent remover.

**② Feature Extraction**

This module extracts the feature vector from the speech. We employed popular features, two spectral features (MFCC: Mel frequency cepstral coefficient, LPC: Linear predictive coding) and two prosodic features (pitch, energy), in existing methods of the speech emotion recognition area [31,32]. The reasons for selecting these features are shown in Table 1.

The process of this module is as follows. At first, speech data is split to 16 ms and then the filter-bank values are extracted, including 13 MFCC, 10 LPC, energy, and pitch in each frame. Then, it calculates the statistical feature vector, which includes the mean, standard deviation, maximum, and minimum. Finally, we use a total of 100 features in the recognition process. Table 1 shows the feature vector scheme description.

**③ Insufficient Data Reinforcement**

This module reinforces the insufficient target emotional samples from an initial constructed multiple user speech dataset when the acquired target user speech samples in a particular emotional label is not enough to train. Regarding reinforcement of the target user training dataset from other users, the overall labeled dataset in a multiple user speech dataset is transformed into an unlabeled statement. Then, we measure the distance from the extracted feature vectors through module 1 and 2 from not only labeled target user speeches, but also unlabeled multiple user speeches. The distance between the unlabeled speech data and the mean value of the acquired target user speech is calculated to measure the similarity. Then, the training dataset is reinforced with the speech that has the most similarities.

**④ Absent Emotion Data Reinforcement**

This module replaces the dataset of empty target emotional samples through similar user emotional speeches from another user speech dataset when some particular emotional label samples were never acquired from the target user. Regarding the similar user emotional speech selection from other users, the distance is measured on each emotional category between the target user and other user through data distribution factors, such as the median, variance, skewness, and kurtosis, for the target user as well as every user in the initial constructed multiple user dataset. Then, the most similar emotion data among the other users is copied to the empty target user emotional label dataset based on the distance from the distribution factors.

**⑤ Heuristic-based Data Selection**

This module selects real cases for the training dataset based on the proper heuristic methods of steps 3 and 4. We designed a heuristic rule considering two kinds of scenarios, which are an insufficient and absent emotion data environment as well as the emotionally-imbalanced samples.

**⑥ SMOTE-based Data Augmentation**

This module builds the final dataset by reinforcing the virtual dataset using the SMOTE algorithm, based on the selected dataset in step 5.

**⑦ Model Creation and Classification**

This module creates a training model based on the boosted dataset from step 6 and then classifies emotions from a new speech input from the target user.

## 3. Proposed Adaptive Data Boosting Methodologies

To provide a personalized model for the target user, it is important to collect a varied amount of target user speech in a balanced manner. However, the target user’s speech may not exist when using the recognition process for the first time, and it is impossible to collect emotion data if the user does not appropriately express themselves during the data collection period. In this initial stage, it is difficult to create a personalized model with high accuracy since there is no speech dataset that includes various cases, thus making it impossible to predict the data distribution of the target user. In order to create a highly personalized training model, it is necessary to reinforce and augment various speech data.

Using the SMOTE algorithm [35] is an efficient way to reinforce and augment different speech cases. SMOTE is a well-known over-sampling technique that can resolve the imbalanced data problem where a particular class is biased. The SMOTE method reduces the gap in the number of samples compared to the majority and minority classes by augmenting the samples of the minority class. However, the main limitation of this method is the cold-start problem, in which there is no accurate data generated when the initial input data are limited numbers. The reason is that SMOTE generates the random data in the nearest boundary of acquired data [36]. In small amounts of data, the boundary area is narrowed. Therefore, it can fall into the overfitting problem and show low accuracy with the new input data. To solve this problem, it is important to acquire enough initial samples before oversampling. Therefore, we propose an ADB method to acquire an initial dataset through data reinforcement and data augmentation to create a personalized model with high accuracy with a minimal number of samples.

ADB reinforces and augments real and virtual data to provide a customized model for target users. ADB consists of insufficient data reinforcement, absent emotion data, heuristic-based data selection, and SMOTE-based virtual data augmentation. The descriptions of the detailed methodologies are given in the following sections.

### 3.1. Insufficient Data Reinforcement

The target user speech data is not always acquired in a sufficient amount to create the personalized emotion recognition model. Especially, the target user emotional samples are collected in limited numbers in the initial stage of personalized emotional speech acquisition. If the personalized model is trained in prime numbers of the target user emotional speech, we cannot achieve a high performance on new input data due to the lack of real case data. The proposed method can overcome the insufficient data problems by adding the similar emotional speech of other users to the training dataset of the personalized model.

**Figure 3:**
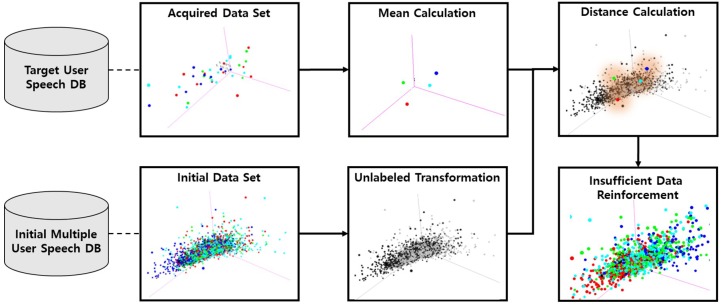
Insufficient data reinforcement workflow.

This section introduces the proposed technique to reinforce insufficient emotional speech of the target users. To increase the amount of insufficient target user emotional speech, the dataset is selected based on the similarity between the target user speech and the multiple-user speech. Figure 3 shows the process of insufficient data reinforcement.

For the similarity calculation between the target user speech and the multiple-user speech, preprocessing and a feature extraction process are performed first, as mentioned in steps 1 and 2 of Section 2. Then, the target user dataset is separated into different emotion classes and the mean value of each feature is obtained for each emotion. The distance between the speech relative to the initial multiple-user speech database is calculated and the target user mean values are obtained. Among this process, the labeled data in the initial multiple-user speech database are transformed into unlabeled data. This means that the label information is ignored in the multiple-user speech database. The reason for using an unlabeled transformation is that emotional expressions are different for each user. For example, if the target user’s anger speech pattern is similar to the happiness pattern from the multiple-user speech database, the system classifies the target user’s anger as happiness. This means that the target user’s particular emotional speech can be similar to different emotional speech in other users’ emotional speech when the acoustic pattern is almost the same. Therefore, we ignore the labeled information in the multiple-user speech database when reinforcing the target user training dataset with other users’ similar speech.

Then, the speech samples from the user closest to the target speech mean value are selected. After that, selected unlabeled data of other users are mapped to the most similar target user emotional label and added to the target user training data set.

The distance is measured using an Euclidean distance measurement [37] between the target user’s mean feature vector and each of the other user’s feature vectors, which is then used to determine the similarity. The following Equations provide the distance measurements:

(1) $$\;means_{ei}=\;\frac{1}{N}\;\overset{N}{\underset{j=1}{\sum }}TFeatureVector_{ji}\;$$

(2) $$\;d\left(means_{ei},\;IDS_{m}\right)=\sqrt{\overset{FN}{\underset{i=1}{\sum }}{\left(means_{ei}-IDS_{i}\right)}^{2}}\;$$

In Equation (1), $means_{ei}\;$ is a two-dimensional array that stores the average value of the acquired target user emotion voice feature vectors, where e is the corresponding emotion index, i is the index of the feature vector, ***N*** is the number of data, j is the index of the data, and $TFeatureVector_{ji}$ is the extracted statistical speech feature vector via the feature extraction module mentioned in step 2 of Section 2. In Equation (2), $d\left(means_{ei},\;IDS_{m}\right)$ represents the distance between two vectors, where  m is the index of the initial multiple-user speech and $IDS_{m}$ is the initial dataset consisting of multiple users. Equation (1) is performed independently for each emotional label of the acquired target user, and Equation (2) is performed based on the results of Equation (1). In the case of the initial dataset, i , in Equation (2), all of the data are retrieved regardless of the label, and then the distance is calculated for each emotion. Finally, the process of sequentially selecting similar data to reinforce the insufficient data according to distance is performed via the following Algorithm 1.

**Algorithm 1** Insufficient Data Reinforcement **Input:**
 TDS(1…N)—Target User Dataset     ***I***DS(1…M)—Initial Multiple User Dataset     ***FN***—Number of Features     ***C***—Number of Classes  **Output: S (1...K)**—Selected Similar Emotional Speeches Dataset  **for *i*** = 1 to ***N* do** $\;TFeatureVector_{i}$ = extractFeatures($TDS_{i})$; $\;{TEmoLabel}_{i}$ = getLabel($TDS_{i})$; **end**  **for *i*** = 1 to ***C* do**   ***cnt* = 0**;   **for *j*** = 1 to ***FN* do**    **for k = 0** to ***N* do**     **if**
$TEmoLabel{\;}_{k}\;$= *i*
**then**      $Tsum_{ij}\;=Tsum_{ij}\;+TFeatureVector_{kj}\;$;      ***cnt* ++**;     **end**    **end**    $Tmeans{\;}_{ij}\;$= $Tsum_{ij}$/**cnt**;   **end** **end**  **for *i*** = 1 to ***C* do**   **for *j*** = 1 to ***M* do**    IFeatureVector = extractFeatures($IDS_{j})$;    $Distances_{ij}$ = *d*($Tmeans_{i},\;IFeatureVector);$   **End** **end**  ***S*** = Sorting (Distances, IDS)**;**  **Return** S;

### 3.2. Absent Emotion Data Reinforcement

Normally, humans do not express different emotions at the same rates in daily life [38]. If the target user does not express a particular emotion for a long time, the training model will be created without any samples for that particular emotional speech. In this case, this particular emotion is not recognized by the system and the accuracy is 0%. We can assume that the target user’s absent emotion data will be similar to that of another user’s emotional speech if they have a similar speech pattern. Based on this assumption, it can be determined that the user is similar if the distribution of the voice data of the target user is similar to the distribution of other user data. Therefore, we propose the reinforcement method to replace the absent target user’s emotion data with the similar user’s emotional speech based on this assumption.

**Figure 4:**
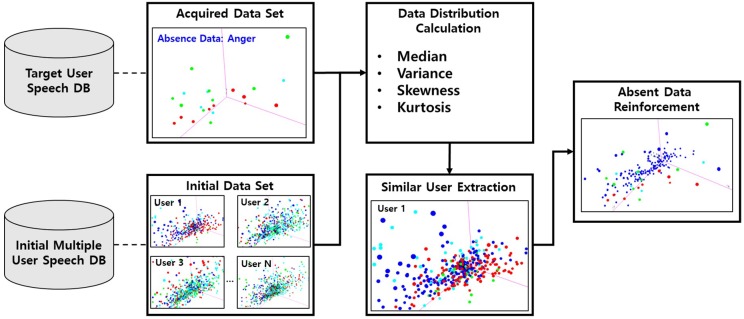
Absent emotion data reinforcement workflow.

This section introduces the proposed method to reinforce data that is not collected from the target user’s particular emotional speech. The proposed method selects the user most similar with the target user from among the emotional speech data of multiple users, and then selects the speech from this similar user. Then, it calculates the distribution similarity based on the speech of each users’ training dataset and selects the most similar user relative to the acquired target user. Finally, this particular absent emotion data will be reinforced regarding the target user’s training dataset considering its similarity with the other user’s emotion speech data. Figure 4 shows the process of absent emotion data reinforcement.

We compute the statistical distribution factors [39], including the median, variance, skewness, and kurtosis, from the speech data of both the target user and the other users considering the speech feature vectors extracted in step 2 of Section 2. Then, the similarity degree between the target user and the other users is calculated. The similarity calculation procedure is the same as in Section 3.1, and the data of the user with the lowest distance value is selected via the following Algorithm 2, where the distance is the sum of the data distribution factors of each user. The contents of the speech feature vector distribution to be considered are as follows.

- **Median**—This variable is used to understand the central value from extracted feature vectors for each emotional labeled speech dataset.
- **Variance**—This variable is used to understand the spreading of the data distribution from extracted feature vectors for each emotional labeled speech data set.
- **Skewness**—This variable is used to understand the direction and extent of the data distribution from extracted feature vectors for each emotional labeled speech data set.
- **Kurtosis**—This variable is used to understand the degree of lean to which the emotional labeled dataset of feature vectors is centered.

**Algorithm 2** Absent Emotion Data Reinforcement **Input: **IDS(1…M)—Initial Multiple User Speeches Dataset    ***CT***—Number of Classes from Target User    TFeatureVector(1…N)—Target User Speeches Feature Vector    TEmoLabelSet(1…C)—Acquired Target User Speeches Label Set    ***NU***—Number of Users    ***TID***—Target User ID  **Output: SU (1...K)**—Selected Similar User Speeches Dataset  **for *i*** = 1 to ***M* do** $IFeatureVector_{i}$ = extractFeatures($IDS_{i})$; ${IEmoLabel}_{i}$ = getLabel($IDS_{i})$; $IUserID{\;}_{i}$ = getUserID ($IDS_{i})$; **end**  **for *i*** = 1 to ***N* do** $TCentroidValues_{i}$ = calculateDistributionFactors ($TFeatureVector_{i})$; **end**  **for *i*** = 1 to ***NU* do**   **for *j*** = 1 to ***CT* do**     **for *k*** = 1 to ***M* do**   **if**
$TEmoLabelSet_{j\;}$**=**
$IEmoLabel_{k}$ and ***i*** = $IUserID_{k}$
**THEN**$ICentroidValues_{i}$ = calculateDistributionFactors ($IFeatureVector_{j});$     **end**   **end** **end**  **for *i*** = 1 to ***NU* do**   **for *j*** = 1 to ***CT* do** $SumDistances_{ij}$ = $SumDistances_{ij}$ + EuclidianDistance($TCentroidValues_{j},\;\;ICentroidValues_{ij});$   **end** **end**  ***US*** = Sorting (SumDistances, IDS);  **Return *US***;

### 3.3. Heuristic-Based Data Selection

In this section, we present a heuristic rule to construct the initial training dataset based on the user-similar speech dataset and the dataset of similar users extracted in Section 3.1 and Section 3.2. We should first define what is meant by a sufficient amount of training data and then determine which data are used to reinforce and create the heuristic rule for selection of the final real speech cases. When defining the required amount of data, we are specifically determining how much of the other users’ data is needed. The reason is that if the system takes only a few data from another user’s speech when lacking target user data, it is difficult to generate an accurate training model. In addition, if the data of the other users is utilized too much, the recognition results are the same as those of using the SD model. Therefore, we set the sufficient data amount as 200 data per each emotion, based on the research results of the data augmentation study [40].

**Figure 5:**
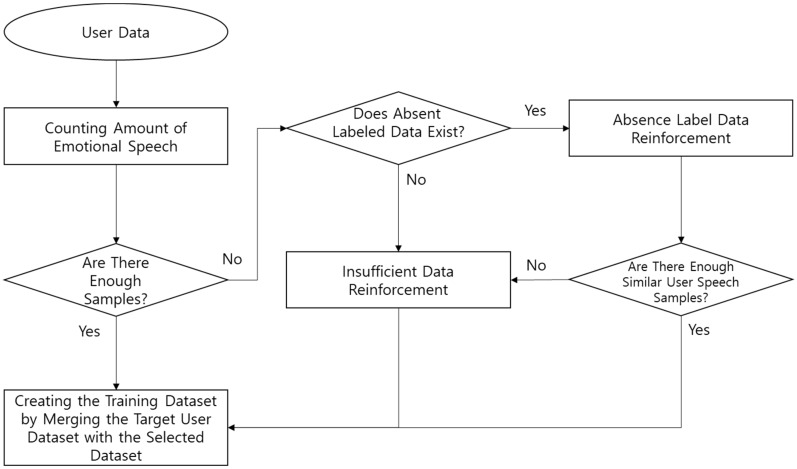
Heuristic-based data selection workflow.

The proposed heuristic rule-based data selection algorithm is composed as follows. When the target user’s emotional speech is input, the system confirms whether the input emotional speech samples are comprised in a sufficient amount of data for each emotion. If a sufficient amount of data is acquired, the emotional dataset is constructed with the customized training dataset. If not, the data reinforcement process will reinforce this data using a sufficient number of samples from another user’s speech pattern. If there is even a single dataset available for a particular emotion, a similar speech is selected through the insufficient data reinforcement process. When the number of samples of the particular emotion is 0, the similar user speech is selected and reinforced via the absent Emotion data reinforcement process. If the selected samples from the absent emotion data reinforcement process are not enough, the system then performs the insufficient data reinforcement process based on the mean values of the particular emotional speech of a similar user. Figure 5 shows a flow chart of the heuristic-based data selection rule.

### 3.4. SMOTE-Based Data Augmentation

SMOTE is the method used to generate the dataset for a minority number of particular class samples in the classification model. At first, SMOTE finds the K nearest neighbors of the minor class samples and finds the difference between the current sample and these K neighbors. This difference is multiplied by a random value between 0 and 1 and is then added to both the training data as well as the original sample. The SMOTE algorithm increases the number of minority classes, which has the smallest number of samples, repeating this several times until the numbers of samples for all classes are balanced. In addition, this algorithm reinforces untrained case data by oversampling this data virtually. This method increases the recognition accuracy of the new input data.

However, the cold-start problem, in which the misrecognition rate increases during the initial stage, occurs when the number of acquired sample data is too small due to the generation of limited ranges of oversampled data, thus it cannot generate accurate samples for the absent emotion data for SMOTE. The cold-start problem of SMOTE can be solved using the dataset extracted from the proposed heuristic-based data selection process. Then, if the data are amplified using SMOTE, the accuracy can be improved even at the initial stage. Therefore, the final training dataset is constructed by reinforcing the virtual case data using the SMOTE algorithm for the training dataset, which is selected via the data reinforcement technique.

## 4. Model Creation and Classification

In this section, we generate the training model using common classification techniques. Choosing an appropriate classifier is important for creating a training model in speech emotion recognition. Machine learning algorithms, such as support vector machines (SVM), decision trees, and random forest, have unique characteristics when generating and recognizing training models.

In this paper, we use a random forest classification algorithm [41] to perform training model generation and recognition. This random forest algorithm was first introduced to mitigate the disadvantages of overfitting and instability common among decision trees. A random forest is a method of creating a single model by combining multiple decision trees. Multiple trees are created by applying randomness to observations and variables. This process generates N bootstrap samples, N trees with arbitrary bootstrap samples and variables, and an ensemble training classifier, which has the advantage of excellent prediction and high stability. Therefore, this classifier is an effective algorithm for speech-based emotion recognition, which can build a reliable training model with few data.

## 5. Experiment

In this section, we introduce the experimental environment and the results. We performed the experiment using IEMOCAP (Interactive Emotional Dyadic Motion Capture) [42], which is a public emotion speech dataset. The IEMOCAP dataset has an extremely large number of data compared to other similar datasets consisting of various speech patterns from real environments. In other recent studies, the five-fold cross validation technique with the four emotions of anger, sadness, happiness, and neutral has shown a low accuracy of about 60%, which has been challenging to overcome [43]. Therefore, the IEMOCAP dataset was selected for our experimental dataset, for which individual datasets are sufficient and clearly exhibit accuracy improvements. In our experimental method, the accuracy of the personalization model generation was calculated by randomly selecting training data and test data from the target user and increasing the number of training data.

### 5.1. Experimental Environment

The purpose of the experiment in this paper is to verify the performance of the personalized emotion recognition model creation method. The proposed method uses the existing SI model when the target user’s data is 0. Since the user data is collected more than once, the training model is rapidly changed by the retraining process using the proposed adaptive data boosting (ADB) method. In order to verify the performance of this technique, the number of personalized data must be enough to be able to train and test.

In the speech emotion recognition area, there are many well organized open datasets, such as eNTEFACE [44], Emo-DB [45], and the Surrey Audio-Visual Expressed Emotion (SAVEE) Database [46]. These databases consist of hundreds to thousands of samples. Most of the existing SI studies used k-fold cross validation when evaluating their algorithm. It means they utilize all data fully to train and test. However, our approach can verify the utilization of an individual target user dataset only to train and test. This means separating training data sets and test data sets to create a personalization model when there are few individual data sets, such as Emo-DB, eNTERFACE, SAVEE, and IEMOCAP, which does not only consider personalization data much, but also has difficulty in measuring accuracy. Therefore, for accurate evaluation, we have required a large amount of individual emotional speech data. Table 2 shows the representation of the existing speech database organization. Existing databases have an insufficient amount of individual emotion data, such as 20 data. These environments have limited choice of user training data and test data, making it difficult to conduct accurate indirect comparison experiments. Finally, we have selected IEMOCAP, which has the largest number of total samples about 100 emotional samples of each emotion per person.

The IEMOCAP dataset is composed of 10,038 corpus samples with 10 labels (neutral, frustration, anger, sadness, happiness, excited, other, surprise, fear, and disgust), which are speech data continually collected through a script. Each sample from the IEMOCAP dataset is annotated with multiple labels from many audiences. We chose a representative label through voting. However, the dataset contains ambiguous emotions, such as excited and frustration. Further, the number of data among surprise, fear, disgust, and other is too small. Therefore, it is difficult to conduct precise experiments when the data is divided into training and test datasets. Table 3 shows the original IEMOCAP dataset structure.

**Figure 6:**
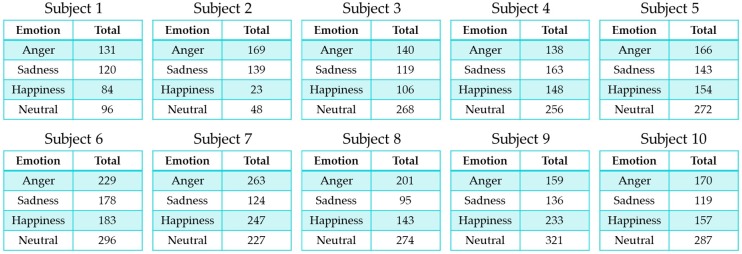
Refined IEMOCAP dataset represented by each user.

Therefore, we transformed the data for the excited and frustration emotion labels to other annotated emotion labels so that these labels are ambiguous and have a high composition ratio in the dataset. We did this by selecting the second most voted label from the IEMOCAP dataset. In addition, we conducted experiments using data for only four emotions: Neutral, anger, sadness, and happiness. Table 4 shows the number and ratio of refined data and Figure 6 shows the number of user-specific samples.

### 5.2. Experimental Methodologies

The traditional emotion recognition experiments were usually conducted using the five-fold cross validation method. This evaluation method yields a high accuracy and includes the target user data in the training dataset, where the number of training data is relatively large. However, this method is not suitable for measuring the performance in personalized emotion recognition experiments, as there is only a small amount of target user training data. Therefore, we aimed to verify the individual accuracy performance using a minimal target user training dataset combined with a new experimental method.

In this new experiment, the training dataset and test dataset were randomly divided without considering the emotion label balance to create an environment similar to real speech acquisition with a limited dataset. At first, we decided the number of maximum training data samples. We allocated the training data and test data to half and half, and we also constructed the sufficient test data samples for evaluation. As a result, we set maximum training data to 300 considering the total number of data is 6925 and the minimum number of data is 379 in subject 2. The remaining data not included in the training dataset were used as the test dataset. Secondly, we incrementally increased the size of the training dataset for each target user starting from a minimum of 50 to a maximum of 300.

**Figure 7:**
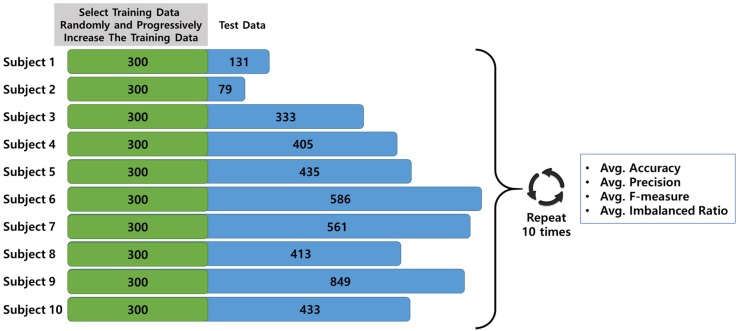
The concept of the experimental methodologies.

This is done to progressively measure the accuracy, precision, and f-measure according to the number of target user training data when creating the personalized training model. Additionally, the average accuracy and precision were measured by repeating the experiment 10 times for fairness. In other words, test data is randomly fixed in each experiment and the training data changes from 50 to 300 incrementally. (e.g., subject 1 had 431 utterances; total dataset: 431, training dataset: 50–300, test dataset: 131). Figure 7 shows the process of the experimental methodologies.

We performed four kinds of comparison evaluation to validate if the proposed method is really efficient in an emotionally imbalanced small sample environment. Furthermore, we also employed the imbalance ratio (IR) [47] to understand how much emotional data is unbalanced and improved. The experiment consists of four criteria as follows.

- Exp. 1—SI (Speaker Independent): The experiment using target user speech data as the test data and creating a training model with the remaining nine users’ datasets. (Standard Model).
- Exp. 2—PM (Personal Model): The experiment conducted by constructing a training model only with personal user speech data.
- Exp. 3—SMOTE: The experiment applying the SMOTE technique alone.
- Exp. 4—Proposed Method: The experiment using the proposed ADB.

### 5.3. Performance Evaluation Results

In this section, we describe the results of the recognition accuracy of the four experiments introduced in Section 5.2. The experiments were performed using implemented Sequential Minimal Optimization (SMO), J48, and random forest in the WEKA Library [48] to estimate which classifier shows the best performance. The WEKA Library is a well-known machine learning open source library. Table 5 shows the average accuracy, weighted average precision, and weighted average f-measure for all four experiments using various classifiers and how many target user data we use to train. In Table 5, the accuracy in every classifier in all experiments is incrementally increased while the target user’s training data is increased. The proposed method (Exp. 4) always shows the highest accuracy among all three classifiers, as well as for all numbers of target user data. In addition, the performance of the random forest classifier used in the proposed framework is the highest.

In the SMO case, we select the RBF kernel, which is normally used in the speech emotion recognition area. The advantage of using the RBF kernel is that it restricts training data to lie in specified boundaries. The RBF kernel nonlinearly maps samples into a higher dimensional space, which means it can handle the case when the relation between class labels and attributes is nonlinear unlike the linear kernel. The RBF kernel has less numerical difficulties than the polynomial kernel [49]. Therefore, we used the RBF kernel for the SVM classifier. Additionally, the parameter of the Gamma and C is set to default values as in the Weka Library (Gamma Value = 0.01, C value = 1). We also used the standardization process in the RBF kernel.

In the experiment using SMO, there is a large difference between the small amount of training data and large amount of training data. As a result, Exp. 1 shows similar accuracy (48.603%) compared with other experiment results when the target user training data is 300 (about 50%). In the personalized experiments results (Exp. 2–4), we can see that the SMO classifier requires lots of target user training data to create a personalized model. That means the SMO classifier using default parameters is more suitable to create a general model than a personalized model. If the Gamma and C value are set to the optimized value, the accuracy can be improved slightly more.

In the experiment using J48, the result of Exp. 1 shows low accuracy (35.178%), and personalized experiments of Exp. 2 and Exp. 3 do not significantly improve the accuracy even though the amount of training data for the target user increases (32.5% to 40%). Exp. 4 shows that the accuracy improves continuously as the target user data increases (35.4% to 55.1%). However, the accuracy is poor in small data environments. This means that the J48 classifier is hard to create a personalized model when the acquired amount of data is small.

In the experiment using random forest, the result of Exp. 1 shows moderate accuracy (42.048%), and the result of Exp. 2 shows that the accuracy improves very slowly (40.8% to 46.3%). The result of Exp. 3 shows the accuracy is increased rapidly (36.6% to 64.5%), and Exp. 4 shows the best accuracy compared with all other experiments (50.9% to 67.6%). Therefore, we know that the random forest classifier is suitable to create a personalized model with our proposed method.

Table 6 shows the status of imbalanced levels represented by the imbalanced ratio (IR) between the majority class and minority class. Exp. 1 means the standard IR value in the IEMOCAP dataset. Exp. 2 does not solve the imbalanced environment over the whole periods, and Exp. 3 solves a little bit in the small amount data environment. Exp. 4 solves the imbalanced data in not only the small data environment, but also the large data environment. The IR measurement is calculated by Equation (3).

(3)  Imbalanced Ratio = Major Class/Minor Class 

**Figure 8:**
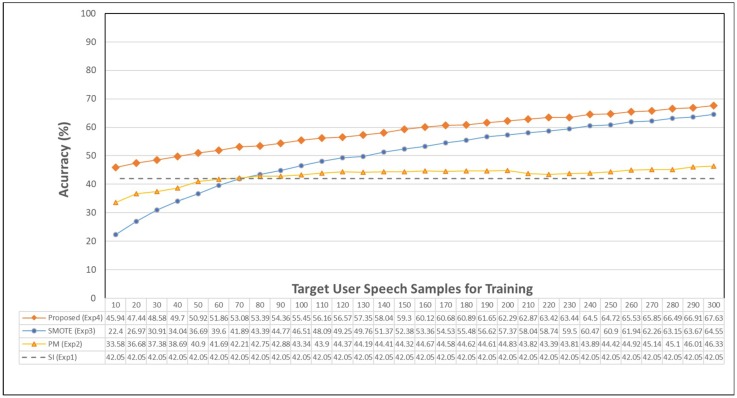
Detailed experimental results of the random forest classifier.

Figure 8 shows the detailed results using the random forest classifier. We can see that the proposed method always shows the highest accuracy.

The experimental results of Exp. 1 show an average of 42.05%. Before the target user speech exceeds 70, the performance is higher than both Exp. 2 and Exp. 3. After that value, however, Exp. 2 and Exp. 3 show a higher accuracy. Exp. 3 shows a lower accuracy than Exp. 2 when the number of target user samples is less than 70. Past this value, Exp. 3 shows a higher accuracy than Exp. 2, where the accuracy difference is about 19% when the number of target user samples is 300. The reason is due to the cold-start problem of SMOTE, where precise oversampling is impossible when the number of target user samples is a limited number, such as 10 to 70. After that, when the target user data is sufficiently acquired, we can see that the accuracy is rapidly increased.

Exp. 4 exhibits high performance across all the experiments over the whole period due to the construction of a sufficient number of data with the proposed ADB method from other users even in the small amount of data environment. The results in the large amount of data environment of Exp. 3 and Exp. 4 are becoming similar, which are influenced by SMOTE that the proposed ADB is also including SMOTE. However, in the small amount of the target user data environment, the result of Exp. 4 clearly shows higher accuracy than Exp. 3, where the accuracy difference is about 23%. In other words, we can see that the proposed ADB method solves the cold-start problem of SMOTE efficiently.

**Figure 9:**
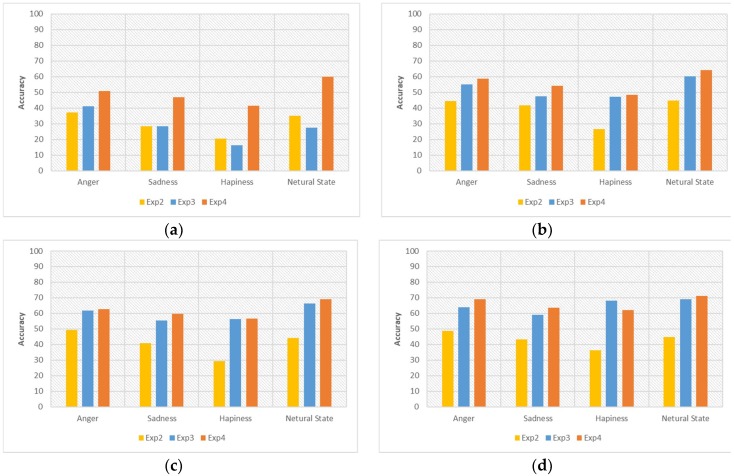
Specific experiment results—(**a**) 10 target user data; (**b**) 100 target user data; (**c**) 200 target user data; (**d**) 300 target user data.

The graph inside Figure 9 shows the recognition accuracies versus the number of target user samples used in training to understand each emotional label accuracy. We can see the recognition accuracy is kept balanced in Figure 9.

In Exp. 2, the accuracy balance is not kept before the target user training data is 200. Especially, the happiness label kept the lowest accuracy. The reason is that Exp. 2 uses only the personal user data in the environment of small imbalanced samples. Therefore, the recognition result shows a quite different accuracy between the most acquired emotional label data and lowest acquired emotional label data.

Exp. 3 did not keep the balanced accuracy when the target user training data was 10. The reason is related to the SMOTE cold-start problem as we have already mentioned. In 10 target user training data, the happiness label has poor accuracy compared with other emotional labels due to the generation of inaccurate data. After acquiring a sufficient amount of target user data, we can see the rapid improvement of accuracy in the happiness label.

Exp. 4 shows more balance and higher accuracy among all of the comparative experiments over the whole period. As a result, we can see that the proposed method can create a more adaptive personalized model in an emotionally imbalanced small samples environment.

## 6. Conclusions

In this paper, we proposed a robust personalized emotion recognition framework considering the small and imbalanced data environment problem in adaptive speech-based emotion recognition. The adaptive data boosting (ADB) technique used in the proposed framework resolves the cold-start problem during the initial recognition stage by creating a customized dataset, merging the acquired target speech with other user speech. By utilizing repetitive individual speaker independent experiments, the proposed method has demonstrated its ability to create a highly accurate training model for a target user, even if there are very small or large numbers of samples. This method effectively generates the target user training model during the initial stage and can incrementally create a training model. We assume that generating a personalized model using the target user’s unlabeled speech, which is acquired in a real-time setting, will show a higher accuracy than using the existing speech data of other users. However, existing public emotion databases have insufficient speech data regarding individual users when trying to create accurate personalized models. Further, the IEMOCAP dataset does not have enough target user speech data to perform the experiment using the unlabeled data of each user. It is possible to generate a more effective personalization model by acquiring unlabeled data from a large number of target users and applying the proposed technique. In our future work, we plan to further study creating a robust personalized model by utilizing the unlabeled dataset of the target user. Additionally, we are also going to conduct additional experiments using state of the art classification methods. Currently, we cannot conduct direct comparison with other studies as the data environment, research goal, and methodologies are quite different. However, we will figure out a solution for this later. Also, we will further conduct research integrating emotional speech databases, such as Emo-DB, eNTERFACE, SAVEE, and IEMOCAP, to validate the generalization of our framework.

## Figures and Tables

**Table 1:** Feature vector scheme description.

Categories	Statistical Values	Number of Features (100)	Description
13 MFCC	- Mean - StdDev - Min - Max	52 (13 × 4)	MFCC is a coefficient, which represents audio, based on the perception of human auditory systems. MFCC has a simple calculation, anti-noise, good ability of distinction, and many other advantages. It is a commonly used feature of speech [33].
10 LPC		40 (10 × 4)	LPC is a tool used mostly in audio signal processing and speech processing for representing the spectral envelope of a digital signal of speech in a compressed form, using the information of a linear predictive model. It is one of the most powerful speech analysis techniques, and one of the most useful methods for encoding [34].
Pitch		4	Pitch and energy are two of the most important features for determining emotion in speech. Individual’s emotional state is strongly related to pitch and energy while pitch and energy of a speech signal expressing happiness or anger is, usually, higher than those associated with sadness [32].
Energy		4	

**Table 2:** Organization of existing emotional speech database.

Emotional Database	Total Samples	Emotions	Speakers	Avg. Samples per Person	Avg. Samples of Each Emotion per Person
Emo-DB	535	7	10	53.5	7.6
eNTERFACE	1166	6	42	27	4.5
SAVEE	480	8	4	120	15
IEMOCAP	10,038	10	10	1003.8	100.3

**Table 3:** Original IEMOCAP dataset structure.

Emotion	Number of Samples	Rate
Anger	1229	12.24%
Sadness	1182	11.78%
Happiness	495	4.93%
Neutral	575	5.73%
Excited	2505	24.96%
Surprise	24	0.24%
Fear	135	1.34%
Disgust	4	0.03%
Frustration	3830	38.16%
Other	59	0.59%
**Total**	**10,038**	**100%**

**Table 4:** Refined IEMOCAP dataset organization.

Emotion	Number of Samples	Rate
Anger	1766	25.51%
Sadness	1336	19.29%
Happiness	1478	21.34%
Neutral	2345	33.86%
**Total**	**6925**	**100%**

**Table 5:** Experimental results for each classifier (unit %).

Classifier	Experiment		Target User Data Samples for Training					
			50	100	150	200	250	300
**SMO (RBF Kernel)**	**Exp. 1**	**Accuracy**	48.603					
		**Precision**	0.512					
		**F measure**	0.478					
	**Exp. 2**	**Accuracy**	37.245	42.257	44.752	47.583	48.823	50.542
		**Precision**	0.293	0.382	0.452	0.474	0.454	0.500
		**F measure**	0.275	0.371	0.335	0.412	0.414	0.474
	**Exp. 3**	**Accuracy**	28.119	35.533	42.018	43.986	46.379	49.569
		**Precision**	0.313	0.453	0.454	0.498	0.510	0.518
		**F measure**	0.197	0.297	0.367	0.390	0.419	0.415
	**Exp. 4**	**Accuracy**	35.421	47.069	49.989	51.736	53.449	55.108
		**Precision**	0.461	0.490	0.523	0.529	0.546	0.559
		**F measure**	0.300	0.438	0.474	0.505	0.523	0.540
**J48**	**Exp. 1**	**Accuracy**	37.291					
		**Precision**	0.3952					
		**F measure**	0.3574					
	**Exp. 2**	**Accuracy**	35.178	37.916	39.784	40.529	40.707	40.027
		**Precision**	0.350	0.390	0.397	0.406	0.409	0.400
		**F measure**	0.328	0.376	0.387	0.398	0.400	0.389
	**Exp. 3**	**Accuracy**	32.586	37.621	39.374	39.657	40.523	41.074
		**Precision**	0.390	0.432	0.425	0.420	0.436	0.425
		**F measure**	0.298	0.386	0.382	0.390	0.412	0.407
	**Exp. 4**	**Accuracy**	36.131	42.268	47.931	53.294	56.542	60.589
		**Precision**	0.399	0.447	0.500	0.540	0.573	0.615
		**F measure**	0.350	0.421	0.481	0.533	0.565	0.607
**Random Forest**	**Exp. 1**	**Accuracy**	42.048					
		**Precision**	0.462					
		**F measure**	0.441					
	**Exp. 2**	**Accuracy**	40.891	43.342	44.324	44.834	44.420	46.329
		**Precision**	0.414	0.421	0.444	0.452	0.444	0.453
		**F measure**	0.412	0.421	0.443	0.450	0.444	0.457
	**Exp. 3**	**Accuracy**	36.692	46.514	52.378	57.362	60.902	64.550
		**Precision**	0.535	0.570	0.590	0.620	0.650	0.669
		**F measure**	0.435	0.513	0.556	0.599	0.632	0.650
	**Exp. 4**	**Accuracy**	50.925	55.448	59.302	62.293	64.722	67.633
		**Precision**	0.503	0.554	0.621	0.658	0.661	0.683
		**F measure**	0.506	0.554	0.612	0.640	0.650	0.680

**Table 6:** Average imbalance ratio for each experiment.

Experiment	Target User Data Samples for Training					
	50	100	150	200	250	300
Exp. 1	1.755					
Exp. 2	5.646	6.074	4.087	4.021	3.188	2.707
Exp. 3	2.914	1.990	1.666	1.730	1.560	1.973
Exp. 4	1.987	1.702	1.560	1.578	1.529	1.519
